# Determination of energy values in pistachio shell powder and soybean hulls fed to gestating and lactating sows

**DOI:** 10.1093/tas/txae135

**Published:** 2024-09-12

**Authors:** Yeonwoo Kim, Su A Lee, Hans H Stein

**Affiliations:** Division of Nutritional Sciences, University of Illinois at Urbana-Champaign, Urbana, IL, USA; Department of Animal Sciences, University of Illinois at Urbana-Champaign, Urbana, IL, USA; Division of Nutritional Sciences, University of Illinois at Urbana-Champaign, Urbana, IL, USA; Department of Animal Sciences, University of Illinois at Urbana-Champaign, Urbana, IL, USA

**Keywords:** digestibility, energy, fiber, pistachio shell powder, sows, soybean hulls

## Abstract

Pistachio shell powder is a high-fiber co-product from the pistachio nut industry that may provide energy and nutrients in animal diets, but no data have been reported for the nutritional value of pistachio shell powder when fed to pigs. Two experiments were, therefore, conducted to test the hypothesis that apparent total tract digestibility (**ATTD**) of gross energy (**GE**), dry matter (**DM**), and total dietary fiber (**TDF**) and concentration of digestible energy (**DE**) in pistachio shell powder are not different from those in soybean hulls when fed to gestating or lactating sows. In experiment 1, 24 gestating sows were housed in metabolism crates and fed a corn-based basal diet or 2 diets that contained corn and 20% pistachio shell powder or corn and 20% soybean hulls. Sows were fed experimental diets for 13 d with feces and urine being quantitatively collected for 4 d after 6 d of adaptation. In experiment 2, 24 lactating sows were housed in farrowing crates and fed a diet based on corn and soybean meal (**SBM**) or 2 diets that contained corn, SBM, and 20% of either pistachio shell powder or soybean hulls, and feces were collected for 6 d after 7 d of adaptation to the diets. Results indicated that for gestating sows, the diet containing soybean hulls had greater (*P* < 0.05) ATTD of DM, GE, and TDF than the diet containing pistachio shell powder. The DE and metabolizable energy (**ME**) in the pistachio shell powder diet were less (*P* < 0.05) than in the basal diet and the diet containing soybean hulls. The ME in pistachio shells (2,606 kcal/kg DM) was less (*P* < 0.05) than in soybean hulls (3,645 kcal/kg DM). When fed to lactating sows, ATTD of DM, GE, and TDF in the diet containing pistachio shell powder was less (*P < *0.05) than in the diet containing soybean hulls or in the basal diet. The DE in the diet containing pistachio shell powder was also less (*P *< 0.05) than in the soybean hulls diet. The DE in pistachio shell powder (1,664 kcal/kg DM) was less (*P *< 0.05) than in soybean hulls (2,795 kcal/kg DM). In conclusion, the ATTD of DM and GE and the DE in pistachio shell powder were less than in soybean hulls, and inclusion in lactation diets, therefore, needs to be limited.

## Teaser

Pistachio shell powder is a high-fiber co-product from the pistachio industry, but when fed to gestating or lactating sows, pistachio shell powder has reduced digestibility of energy compared with soybean hulls.

## INTRODUCTION

California is the leading producer of pistachios in the United States and globally followed by Iran and Turkey (Toghiani et al., 2023). Production of pistachios is increasing, and the American Pistachio Growers Association estimated that the production would reach 1.04 million metric tons in 2031 (Tootelian, 2023). As a result, around 15.6 thousand metric tons of pistachio shells will be produced after the nuts are processed for human consumption (Tootelian, 2023). Whereas pistachio nuts are a good source of protein, with a Digestible Indispensable Amino Acid Score greater than 75 (Bailey and Stein, 2020), pistachio shells are not used for human consumption. The inclusion of a pistachio shell powder product in diets fed to sheep had no negative impact on apparent total tract digestibility (**ATTD**) or nitrogen retention (Ghasemi et al., 2012), but no data for feeding pistachio shells to pigs have been reported. However, if pistachio shells can be finely ground, the shell powder can be used as a high-fiber ingredient in diets for pigs. Specifically, gestating sows may benefit from the high-fiber concentration in pistachio shell powder because this may increase satiety and reduce stress, but at this time, there is no information about the nutritional value of pistachio shell powder when fed to sows. If the nutritional value of pistachio shell powder can be established, this ingredient can potentially be used in diets for sows as an alternative to other high-fiber ingredients. Therefore, 2 experiments were conducted to test the null hypothesis that ATTD of gross energy (**GE**), dry matter (**DM**), total dietary fiber (**TDF**), and digestible energy (**DE**) in pistachio shell powder are not different from those in soybean hulls when fed to gestating or lactating sows.

## MATERIALS AND METHODS

Protocols for 2 experiments were submitted to the Institutional Animal Care and Use Committee at the University of Illinois and approved prior to initiation of the experiments. In both experiments, Camborough sows (PIC, Hendersonville, TN, USA) were used.

One batch of pistachio shell powder (The Wonderful Company, Los Angeles, CA, USA, and the University of Kansas, Lawrence, KS, USA) and one batch of soybean hulls (South Central FS, Watson, IL, USA) were procured and the same sources of the 2 ingredients were used in both experiments (Table 1). Likewise, the same source of corn, which was grown locally, was used in both experiments.

**Table 1.** Analyzed nutrient composition of feed ingredients, as-fed basis

Item, %	Corn	Soybean meal	Pistachio shell powder	Soybean hulls
Dry matter	86.78	89.44	96.30	90.87
Gross energy, kcal/kg	3,796	4,257	4,379	3,806
Ash	0.95	6.15	0.29	5.05
Crude protein	6.03	45.16	1.69	11.16
Acid hydrolysis ether extract	3.36	2.13	1.72	4.26
Starch	61.88	3.60	ND^1^	0.23
Total dietary fiber	13.3	29.9	93.1	71.2
Soluble dietary fiber	2.8	8.6	1.2	11.3
Insoluble dietary fiber	10.5	21.2	91.9	59.9
Soluble nonstarch polysaccharide				
Rhamnose	ND	0.03	0.02	0.30
Fucose	ND	0.03	ND	0.01
Arabinose	0.11	0.15	0.05	0.51
Xylose	0.06	ND	ND	ND
Mannose	0.16	0.31	0.27	2.06
Galactose	ND	0.29	0.09	1.15
Insoluble nonstarch polysaccharide				
Rhamnose	ND	0.19	0.32	0.37
Fucose	ND	0.29	ND	0.21
Arabinose	1.37	2.12	0.48	3.86
Xylose	2.10	1.12	35.07	7.38
Mannose	0.14	0.71	0.10	3.32
Galactose	0.40	4.38	0.44	1.82
Glucose	0.95	0.31	1.65	1.74
Cellulose	1.90	3.42	25.78	30.99
Indispensable amino acids				
Arginine	0.32	3.45	0.13	0.68
Histidine	0.19	1.28	0.03	0.34
Isoleucine	0.24	2.38	0.06	0.56
Leucine	0.72	3.73	0.09	0.90
Lysine	0.25	3.03	0.08	0.87
Methionine	0.14	0.66	0.02	0.14
Phenylalanine	0.30	2.49	0.06	0.53
Threonine	0.23	1.83	0.06	0.47
Tryptophan	0.05	0.70	<0.02	0.06
Valine	0.32	2.46	0.08	0.62
Total	2.76	22.01	0.63	5.17
Dispensable amino acids				
Alanine	0.46	2.04	0.07	0.55
Aspartic acid	0.44	5.48	0.14	1.28
Cysteine	0.14	0.67	0.03	0.23
Glutamic acid	1.13	8.65	0.21	1.62
Glycine	0.27	1.99	0.07	0.91
Proline	0.55	2.44	0.10	0.67
Serine	0.28	1.94	0.07	0.58
Tyrosine	0.20	1.74	0.02	0.44
Total	3.47	24.95	0.71	6.28
Total amino acids	6.23	46.96	1.34	11.45
Minerals, %				
Ca	<0.01	0.34	<0.01	0.86
P	0.04	0.73	<0.01	0.11
K	0.04	1.93	0.05	0.85
Mg	0.11	0.27	0.02	0.17
Na	<0.01	0.02	0.01	0.03

^1^ND, not detectable.

### Experiment 1: Gestating Sows

Twenty-four gestating sows (initial body weight [**BW**]: 209.85 ± 15.26 kg; parity 2 to 6) that were approximately 65 d into gestation were allotted to 2 blocks of 12 sows using a randomized complete block design with 3 diets and 4 sows per treatment in each block for a total of 8 replicate sows per diet. The breeding group was the blocking factor. A corn-based basal diet and 2 diets containing corn and 20% pistachio shell powder, or corn and 20% soybean hulls were formulated (Table 2). Vitamins and minerals were included in all diets to meet or exceed requirements (NRC, 2012). Daily feed allowance was 1.5 times the maintenance energy requirement for gestating sows (i.e., 100 kcal/kg BW^0.75^; NRC, 2012). Sows had free access to water at all times throughout the experiment. Daily feed allotments were divided into 2 equal meals that were provided at 0800 and 1600 hours.

**Table 2.** Ingredient and nutrient composition of experimental diets, as-fed basis

Item	Experiment 1 (gestating sows)			Experiment 2 (lactating sows)		
	Basal diet	Pistachio shell powder	Soybean hulls	Basal diet	Pistachio shell powder	Soybean hulls
Ingredient, %						
Ground corn	96.47	76.75	76.75	71.73	56.81	57.02
Soybean meal	—	—	—	25.00	19.80	19.87
Pistachio shell powder	—	20.00	—	—	20.00	—
Soybean hulls	—	—	20.00	—	—	20.00
Ground limestone	0.98	0.65	0.65	0.71	0.54	0.36
Dicalcium phosphate	1.65	1.70	1.70	1.26	1.55	1.45
Sodium chloride	0.40	0.40	0.40	0.40	0.40	0.40
Vitamin–mineral premix^1^	0.50	0.50	0.50	0.50	0.50	0.50
Titanium dioxide	—	—	—	0.40	0.40	0.40
Analyzed nutrient composition						
Dry matter, %	87.78	89.41	88.09	87.68	89.62	88.28
Gross energy, kcal/kg	3,716	3,828	3,704	3,798	3,882	3,755
Crude protein, %	6.55	5.60	7.58	16.64	12.84	15.51
Ash, %	4.37	3.88	4.96	5.01	5.04	6.17
Total dietary fiber, %	12.90	29.20	24.00	15.50	32.70	27.40

^1^The vitamin-micromineral premix provided the following quantities of vitamins and micro minerals per kg of complete diet: vitamin A as retinyl acetate, 10,622 IU; vitamin D_3_ as cholecalciferol, 1,660 IU; vitamin E as _DL_-alpha-tocopherol acetate, 66 IU; vitamin K as menadione nicotinamide bisulfate, 1.40 mg; thiamin as thiamine mononitrate, 1.08 mg; riboflavin, 6.49 mg; pyridoxine as pyridoxine hydrochloride, 0.98 mg; vitamin B_12_, 0.03 mg; _D-_pantothenic acid as _D-_calcium pantothenate, 23.2 mg; niacin, 43.4 mg; folic acid, 1.56 mg; biotin, 0.44 mg; Cu, 20 mg as copper chloride; Fe, 123 mg as iron sulfate; I, 1.24 mg as ethylenediamine dihydriodide; Mn, 59.4 mg as manganese hydroxychloride; Se, 0.27 mg as sodium selenite and selenium yeast; and Zn, 124.7 mg as zinc hydroxychloride.

Gestating sows were housed in metabolism crates (0.91 × 2.08 m) that were equipped with a self-feeder, a nipple drinker, and a fully slatted T-bar floor. A screen floor and a urine pan were installed under the T-bar floor for a separate collection of feces and urine. Sows were fed experimental diets for 13 d. The initial 6 d in the metabolism crates were considered the adaptation period to the crates and the diets, and urine and feces were collected from the diet provided during the following 4 d using the marker-to-marker procedure (Adeola, 2001). The fecal collection was initiated when the first marker (i.e., chromic oxide), which was fed in the morning meal on day 7, appeared in the feces, and collection of feces ceased when the second marker (i.e., ferric oxide), which was fed in the morning meal on day 11, appeared. Collected fecal samples were stored at −20 °C immediately after collection. Urine was collected in buckets placed under the urine pans and 50 mL of 3 *N* HCl was added to each bucket every day. Buckets were emptied daily, the weight of the collected urine was recorded, and 10% of the collected urine was stored at −20 °C until subsampling.

### Experiment 2: Lactating Sows

Twenty-four multiparous sows in lactation (initial BW: 228.52 ± 20.07 kg; parity 2 to 6) were used in a randomized complete block design with 2 blocks of 12 sows, 3 diets, and 4 sows per diet in each block for a total of 8 replicate sows per treatment. The breeding group was the blocking factor. The basal diet was formulated based on corn and soybean meal (**SBM**) and this diet met all nutrient requirement estimates for lactating sows (Table 2; NRC, 2012). Two additional diets were formulated by including 20% of either pistachio shell powder or soybean hulls in the basal diet at the expense of corn and SBM. All diets contained corn and SBM at a ratio of 2.87:1.00, and 0.40% TiO_2_ was included in all diets as an indigestible marker.

The lactating sows used in experiment 2 were different from the gestating sows used in experiment 1. Sows were fed a commercial diet for gestating sows before being moved to farrowing crates 7 d before farrowing where they remained until weaning on day 20 post-farrowing. The farrowing crates had fully slatted floors. A commercial lactation diet was provided from day 7 before farrowing to day 4 post-farrowing, but experimental diets were fed from day 5 post-farrowing to day 18 post-farrowing. The initial 8 d were the adaptation period to experimental diets, and fecal samples were collected once daily via grab sampling from days 13 to 18. Collected fecal samples were immediately stored at −20 °C. Experimental diets were provided on an ad libitum basis and water was available at all times.

### Chemical Analysis

At the conclusion of experiment 1, urine samples were thawed and mixed, and a subsample was lyophilized before analysis (Kim et al., 2009). Fecal samples from both experiments were thawed and dried at 55 °C in a forced-air drying oven for 7 d (Heratherm OMH750; Thermo Fisher 1873 Scientific Inc., Waltham, MA, USA). Samples were then ground through a 1-mm screen using a hammermill (model: MM4; Schutte Buffalo, NY, USA), mixed, and subsampled for analysis.

Ingredient, diet, and fecal samples were analyzed for DM (method 930.15; AOAC Int., 2019). Diets and ingredient samples, fecal samples from both experiments, and lyophilized urine samples from experiment 1 were analyzed for GE on an isoperibol bomb calorimeter (Model 6400, Parr Instruments, Moline, IL, USA) using benzoic acid as the internal standard. All diets and ingredients were also analyzed for ash (method 942.05; AOAC Int., 2019), and ingredients, diets, and fecal samples were analyzed for insoluble dietary fiber (**IDF**), and soluble dietary fiber (**SDF**) according to method 991.43 (AOAC Int., 2019) using the Ankom^TDF^ Dietary Fiber Analyzer (Ankom Technology, Macedon, NY, USA). TDF was calculated as the sum of IDF and SDF. Nitrogen was analyzed in ingredients and diets by combustion using a LECO FP628 Nitrogen Analyzer (LECO Corp., Saint Joseph, MI, USA; method 990.03; AOAC Int., 2019) and crude protein was calculated as N × 6.25. Crude fat was analyzed in ingredients by acid hydrolysis using 3 *N* HCl (AnkomHCl, Ankom Technology) followed by crude fat extraction using petroleum ether (method 2003.06; AOAC Int., 2019) on an Ankom fat analyzer (AnkomXT15, Ankom Technology). Titanium was analyzed in lactation diets and feces from lactating sows (method 985.01 A, B, and C; AOAC Int., 2019) using inductively coupled plasma-optical emission spectrometry (**ICP-OES**; Avio 200, PerkinElmer, Waltham, MA, USA). Sample preparation included dry ashing at 600 °C for 4 h (method 942.05; AOAC Int., 2019) and wet digestion with sulfuric acids (method 3050 B; U.S. Environmental Protection Agency, 2000). Minerals were also analyzed in ingredients by ICP-OES. Ingredients were also analyzed for AA (method 982.30 E [a, b, c]; AOAC Int., 2019) on a Hitachi Amino Acid Analyzer, Model No. L8800 (Hitachi High Technologies America, Inc.; Pleasanton, CA, USA) using ninhydrin for postcolumn derivatization and nor-leucine as the internal standard.

Ingredients were analyzed for nonstarch polysaccharides using gas-liquid chromatography based on the individual sugar constituents as alditol acetates after a 3-parallel extraction procedure: 1) total nonstarch polysaccharides, 2) noncellulosic polysaccharides, and 3) insoluble noncellulosic polysaccharides. All procedures followed those described by Jaworski et al. (2015). Total starch was analyzed in ingredients by the amyloglucosidase-alpha-amylase procedure corresponding to the enzymatically hydrolyzed starch converted to glucose, and glucose was quantified by spectrophotometry (method 996.11; AOAC Int., 2019).

### Calculations

Values for ATTD of DM, GE, and TDF, and the concentration of DE were calculated for each diet for both gestating and lactating sows, and the ME in the diets fed to gestating sows was also calculated (Adeola, 2001). By subtracting the GE contributed by corn (experiment 1) or corn and SBM (experiment 2) from GE in diets containing pistachio shell powder or soybean hulls, the DE and ME in pistachio shell powder and soybean hulls were calculated by difference for gestating sows (Adeola, 2001) and the DE was calculated for lactating sows.

### Statistical Analysis

Data were analyzed using the MIXED Procedure of SAS (SAS Inst. Inc., Cary, NC, USA). The homogeneity of the variances among treatments was confirmed. Outliers of the variances were tested using the UNIVARIATE procedure of SAS, but no outliers were detected in either experiment. Sow was the experimental unit for all analyses. For both experiments, the statistical models included diet or ingredient as the fixed effect and block and replicate within block as the random effects. Least square means were calculated, and means were separated with the pdiff option using Tukey’s adjustment if the model *P*-value was significant (Tukey, 1977). Statistical significance was considered at *P *< 0.05.

## Results

Sows remained healthy during both experiments and feed refusals were not observed. All sows assigned to experimental diets in both experiments completed the experiments.

### Experiment 1: Gestating Sows

Feed intake, GE intake, and TDF intake by sows fed the 2 diets containing pistachio shell powder or soybean hulls were greater (*P* < 0.05) than by sows fed the basal diet (Table 3). Weight of feces, GE fecal output, concentration of TDF in feces, and TDF fecal excretion from sows fed the pistachio shell powder diet were greater (*P* < 0.05) than from sows fed the other diets, and sows fed the soybean hulls diet had greater (*P* < 0.05) fecal output, GE output in feces, and fecal excretion of TDF than sows fed the basal diet. Weight of urine was greater (*P *< 0.05) from sows fed the basal diet than from those fed the pistachio shell powder diet with an intermediate value for sows fed the soybean hulls diet, but the magnitude of the difference was greater between basal and soybean hull diets than between the soybean hull and pistachio shell powder diets. However, GE output in urine was not different among treatments. The ATTD of DM and GE was less (*P *< 0.05) in the diet containing pistachio shell powder than in the basal diet or the diet containing soybean hulls, but ATTD of TDF was greater (*P *< 0.05) in the diet containing soybean hulls than in the basal diet, whereas the ATTD of TDF was least (*P *< 0.05) in the diet containing pistachio shell powder. Concentration of DE in the basal diet was greater (*P* < 0.05) than in the other 2 diets and the soybean hulls diet contained more (*P *< 0.05) DE than the pistachio shell power diet. Concentration of ME in the basal diet was also greater (*P *< 0.05) compared with the pistachio shell powder diet, but there was no difference in ME between the basal diet and the soybean hulls diet.

**Table 3.** Apparent total tract digestibility of dry matter, gross energy, total dietary fiber, and digestible energy and metabolizable energy in experimental diets fed to gestating sows^1^ (experiment 1), as-fed basis

Item, %	Basal diet	Pistachio shell powder	Soybean hulls	SEM	*P*-value
Intake					
Diet, kg/d	2.51^b^	2.78^a^	2.75^a^	0.05	0.004
GE, Mcal/d	9.33^b^	10.65^a^	10.18^a^	0.20	0.001
TDF, kg/d	0.32^c^	0.81^a^	0.66^b^	0.01	<0.001
Fecal excretion					
Dry feces output, kg/d	0.16^c^	0.35^a^	0.20^b^	0.01	<0.001
GE, kcal/d	662^c^	1,526^a^	829^b^	41	<0.001
TDF, %	33.17^c^	74.26^a^	36.76^b^	0.87	<0.001
TDF, kg/d	0.06^c^	0.26^a^	0.07^b^	0.01	<0.001
Urine excretion					
Urine output, kg/d	8.87^a^	3.20^b^	5.03^ab^	1.44	0.032
GE, kcal/d	214	241	198	23	0.433
ATTD of DM, %	92.93^a^	86.23^b^	91.98^a^	0.33	<0.001
ATTD of GE, %	92.90^a^	85.69^b^	91.84^a^	0.34	<0.001
ATTD of TDF, %	82.94^b^	67.92^c^	88.68^a^	0.85	<0.001
Energy in diets, kcal/kg					
DE	3,452^a^	3,280^c^	3,402^b^	13	<0.001
ME	3,366^a^	3,194^b^	3,330^a^	15	<0.001

^a-c^Within a row, means without a common superscript differ (*P* < 0.05).

^1^Each least square mean represents 8 observations per diet.

Concentrations of DE and ME on an as-fed basis as well as on a DM basis were less (*P *< 0.05) in pistachio shell powder compared with corn or soybean hulls (Table 4). On an as-fed basis, soybean hulls contained less (*P *< 0.05) DE than corn and on a DM basis, DE and ME in soybean hulls were also less (*P* < 0.05) than in corn. The DE:GE and the ME:GE in corn were greater (*P* < 0.05) than in soybean hulls, and DE:GE and ME:GE were greater (*P *< 0.05) in soybean hulls than in pistachio shell powder.

**Table 4.** Digestible energy (DE) and Metabolizable energy (ME) in corn, pistachio shell powder, and soybean hulls fed to gestating sows^1^ (experiment 1)

Item, %	Corn	Pistachio shell powder	Soybean hulls	SEM	*P*-value
As-fed basis, kcal/kg					
DE	3,578^a^	2,669^c^	3,277^b^	56	<0.001
ME	3,490^a^	2,580^b^	3,258^a^	66	<0.001
Dry matter basis, kcal/kg					
DE	4,076^a^	2,699^c^	3,665^b^	62	<0.001
ME	3,976^a^	2,606^c^	3,645^b^	73	<0.001
Digestibility and metabolizability					
DE:gross energy	94.26^a^	60.96^c^	86.10^b^	1.30	< 0.001
ME:DE	97.53	96.63	99.45	1.25	0.287
ME:gross energy	91.93^a^	58.92^c^	85.61^b^	1.53	<0.001

^a-c^Within a row, means without a common superscript differ (*P* < 0.05).

^1^Each least square mean represents 8 observations per diet.

### Experiment 2: Lactating Sows

Feed intake and GE intake by sows fed the basal diet or the diet containing pistachio shell powder were greater (*P* < 0.05) than by sows fed the diet containing soybean hulls, but there were no differences in feed intake between sows fed the basal diet and sows fed the diet containing pistachio shell powder (Table 5). The ATTD of DM and GE and the concentration of DE in the basal diet was greater (*P* < 0.05) than in the other diets, and the diet containing soybean hulls had greater (*P* < 0.05) ATTD of GE and DM and greater (*P *< 0.05) DE than the diet containing pistachio shell powder. The ATTD of TDF was greater (*P *< 0.05) in the diet containing soybean hulls followed by the basal diet and the diet containing pistachio shell powder. The concentration of DE in pistachio shell powder on an as-fed as well as on a DM basis and DE:GE was less (*P *< 0.05) than in soybean hulls.

**Table 5.** Apparent total tract digestibility of dry matter (DM), gross energy (GE), total dietary fiber (TDF), and concentration of digestible energy (DE) in experimental diets and ingredients fed to lactating sows^1^ (experiment 2)

Item, %	Basal diet	Pistachio shell powder	Soybean hulls	SEM	*P*-value
Intake					
Diet, kg/d	5.70^a^	5.94^a^	4.23^b^	0.33	0.003
GE, Mcal/d	21.65^a^	23.05^a^	15.87^b^	1.27	0.002
TDF, kg/d	0.88	1.94	1.16	0.08	<0.001
ATTD of DM, %	88.77^a^	77.19^c^	85.49^b^	0.59	<0.001
ATTD of GE, %	88.02^a^	75.97^c^	84.16^b^	0.65	<0.001
ATTD of TDF, %	76.77^b^	59.61^c^	81.86^a^	1.54	<0.001
DE in diet, kcal/kg, as-fed basis	3,343^a^	2,951^c^	3,181^b^	25	<0.001
DE in ingredient, kcal/kg					
As-fed basis	—	1,603	2,540	132	<0.001
DM basis	—	1,664	2,795	144	<0.001
DE:GE	—	34.59	68.84	3.48	<0.001

^a-c^Within a row, means without a common superscript differ (*P* < 0.05).

^1^Each least square mean represents 8 observations per diet.

## Discussion

The GE in all diets used in the 2 experiments was in agreement with calculated values, which indicated that errors in diet mixing, subsampling, and GE analysis were minimal. The GE in corn and soybean hulls was less than some previous values (NRC, 2012), which may be because both ingredients contained less protein than previously reported. Although crude protein in SBM was also lower than reported (NRC, 2012), GE in SBM was within the range of previous values (NRC, 2012). The ATTD of GE and concentrations of DE and ME in corn used in experiment 1 were greater than values obtained with growing pigs (NRC, 2012), which is likely a result of the greater energy digestibility by gestating sows compared with growing pigs (Le Goff and Noblet, 2001; Casas and Stein, 2017). The ATTD of GE and concentration of DE in the basal diet fed to lactating sows in experiment 2 were in agreement with calculated values for a corn-SBM diet fed to growing pigs (NRC, 2012), which is likely because lactating sows, like growing pigs, are allowed ad libitum intake of feed, and therefore, have increased feed intake and greater passage rate, compared with gestating sows (Kim et al., 2007).

High-fiber co-products from the human food industry are often used in diets fed to livestock (Zijlstra and Beltranena, 2013; Stein et al., 2015). This is also true for co-products from the nut industry and pecan shells have successfully been incorporated into diets for finishing pigs (Flores et al., 2023) and gestating sows (Buenabad et al., 2022). Likewise, almond hulls can be included in diets for growing pigs without negative impacts on growth performance or nutrient digestibility (Ahammad et al., 2024). However, the main co-product from the production of pistachio nuts, pistachio shells, has until now primarily been limited to industrial uses (Toghiani et al, 2023), although a lower-fiber pistachio co-product may replace lucerne hay in diets for sheep without negatively impacting ATTD of energy or nutrients (Ghasemi et al., 2012). Whereas almond hulls contain around 44% TDF (Fanelli et al., 2023), the pistachio shell powder used in this experiment contained more than 90% TDF resulting in diets with greater total TDF concentrations than if almond hulls or other co-products from the nut industry are used. Pistachio shell powder is, therefore, a unique feed ingredient and to the best of our knowledge, no information about using pistachio shell powder in diets for pigs has been reported. There are, however, possible benefits of including high-fiber ingredients in diets for reproducing swine (Jo and Kim, 2023), and pistachio shell powder was, therefore, included in diets for sows in the current experiments.

Although pistachio shell powder contained more than 90% TDF, almost all the TDF was insoluble, which is the reason the concentration of soluble monosaccharides was very low. In contrast, cellulose made up almost 26% of the ingredients, and xylose contributed 35% of the pistachio shell powder, which was much more than the other ingredients used in the experiment. The high xylose concentration indicates that the fiber in pistachio shell powder likely contains xylan polysaccharides, but these do not appear to include arabinoxylans because the concentration of arabinose was very low.

The relatively high DE that was determined in pistachio shell powder when fed to gestating sows, despite the very high concentration of IDF and TDF in the ingredient, indicates that it is possible to use this ingredient in diets for sows. The greater DE determined in gestating sows compared with lactating sows is likely a result of the restricted feed intake of gestating sows that allows more time for nutrient and energy digestion and absorption and microbial fermentation in the intestinal tract. The lower DE and ME in pistachio shell powder than in soybean hulls is likely not a problem when fed to gestating sows because of the restriction of feed intake of gestating sows. In fact, feeding pistachio shell powder to gestating sows may be an advantage because feeding diets that are high in fiber increases satiety compared with low-fiber diets (Li, 2014). Indeed, the observation that urine excretion from gestating sows fed the pistachio shell powder diet was much less than from sows fed the basal diet indicates that sows fed the pistachio shell powder diet likely were less stressed than sows fed the basal diet because sows with increased stress may spend time playing with the water nipple, which may increase estimated urine output due to water spillage (Li, 2014). The observation that total energy excretion in urine was not different among treatments further indicates that the extra urine output from sows fed the basal diet likely was caused by water spillage as a result of increased stress of the sows. The fact that no negative effects on feed intake were observed when sows were fed the diet containing pistachio shell powder compared with sows fed the control diet further indicates that pistachio shell powder may be used by gestating sows. This may be because the gestating sows were fed a restricted amount of feed, but the possibility that more than 20% pistachio shell powder can be included in diets for gestating sows needs to be investigated in the future. Because the inclusion of dietary fiber in diets for gestating sows sometimes increases the reproductive performance of sows (Jo and Kim, 2023), research to determine the impact of pistachio shell powder on the reproductive performance of sows also needs to be conducted.

The fact that lactating sows fed the diet containing pistachio shell powder had daily voluntary feed intake that was not different from that of sows fed the control diet indicates that sows were not able to compensate for the reduced DE in pistachio shell powder by increasing feed intake, which may be due to gut fill. The daily DE intake will, therefore, be reduced if pistachio shell powder is included in diets for lactating sows, and as a consequence, the inclusion of pistachio shell powder in lactation diets likely needs to be limited. Nevertheless, because the DE in pistachio shell powder was less than in soybean hulls when fed to both gestating and lactating sows, the null hypothesis for the experiment was rejected.

The observation that the ATTD of TDF in diets fed to both gestating and lactating sows was close to or greater than 60% indicates that sows are able to ferment a significant part of the TDF in pistachio shell powder, which is important because more than 90% of the ingredient is TDF. As a consequence, the majority of the energy generated from pistachio shell powder was absorbed in the form of volatile fatty acids that were synthesized as a result of the fermentation of fiber in the hindgut of sows.

Although it is recognized that fecal material analyzed as TDF may contain microbial matter, which results in reduced calculated values for ATTD of TDF (Cervantes-Pahm et al., 2014; Montoya et al., 2015, 2016), this is primarily a problem in diets with low concentrations of dietary fiber, whereas ATTD of TDF in diets with greater concentration of dietary fiber are much less influenced by microbial matter (Cervantes-Pahm et al., 2014). Because of the high concentrations of TDF in the diets containing pistachio shell powder, it is, therefore, unlikely that microbial matter greatly influenced the calculated values for ATTD of TDF.

The feed intake of lactating sows fed the diet containing soybean hulls was less than for the other diets, which is likely because soybean hulls contained more than 10% SDF and SDF has a high water-binding capacity, which results in more gut fill and reduced rate of passage (Tan et al., 2017). However, SDF is more fermentable than IDF (Urriola et al., 2010; Jaworski and Stein, 2017), which is likely the reason the ATTD of TDF was greater in the soybean hulls diet than in the pistachio shell powder diet, despite the greater TDF intake from the pistachio shell powder diet compared with the soybean hulls diet. The fact that even though the ATTD of TDF in diets containing soybean hulls was also greater than in the basal diets is a result of the very low concentration of SDF in corn fiber (Jaworski et al., 2015).

The ATTD of GE and the DE in soybean hulls fed to both gestating and lactating sows in this experiment were greater than values obtained with growing pigs (NRC, 2012; Jaworski and Stein, 2017; Rodriguez et al., 2020). Because sows have a greater capacity to utilize energy from fiber than growing pigs due to their larger intestinal tract and increased microbial fermentation (Casas and Stein, 2017), it appears that energy digestibility and DE are increased in soybean hulls fed to sows compared with growing pigs, but because the diets used in this experiment were not fed to growing pigs, we cannot confirm this hypothesis. It is possible that nutrient digestibility changes throughout gestation because digestibility and retention of Ca and P changes from the first to the second or third trimester of gestation (Kemme et al., 1997; Lee et al., 2019). However, we are not aware of data demonstrating differences in DM and energy digestibility during gestation, and we, therefore, consider energy digestibility obtained from sows in mid-gestation to be representative of the entire gestating period.

It was not the objective to compare data for gestating and lactating sows, but the observation that ATTD of GE and DE in diets fed to gestating sows were greater compared with lactating sows was in agreement with previous data (Acosta et al., 2024). It is possible that differences in feeding methods (i.e., restricted vs. ad libitum) resulted in these differences because the passage rate is increased by greater feed intake, which may result in reduced digestibility (Kim et al., 2007). However, both gestating and lactating sows were fed amounts close to what sows consume on commercial farms, which indicates that these data can also be applied when formulating diets for sows in commercial settings.

In addition to the increased SDF in the soybean hulls used in this experiment, the concentration of fat was also greater than previously reported, which likely also contributed to the increased DE in the soybean hulls because fat not only has a high energy value, but also reduces passage rate in the intestinal tract, and therefore, increases time for digestion and absorption of nutrients (Cervantes-Pahm and Stein, 2008; Zhou et al., 2017). The combination of more SDF and reduced passage rate, however, negatively impacts feed intake of animals allowed ad libitum access to feed as was observed for the lactating sows in this experiment. Due to this reduction in feed intake, the inclusion of soybean hulls in diets for lactating sows needs to be limited, whereas the use of soybean hulls in diets for gestating sows may be advantageous because the reduced passage rate may increase satiety in sows.

In conclusion, DE in pistachio shell powder was 2,699 and 1,664 kcal/kg DM when fed to gestating sows and lactating sows, respectively, which was less than the DE in soybean hulls (3,665 and 2,795 kcal/kg DM, respectively). The ME in pistachio shell powder (i.e., 2,606 kcal/kg DM) was also less than in soybean hulls (i.e., 3,645 kcal/kg DM) when fed to gestating sows. Pistachio shell powder had a high concentration of IDF, which resulted in low energy digestibility and DE and therefore limited the inclusion in diets for lactating sows. Further research is needed to determine whether greater inclusion rates can be used, and the impact of pistachio shell powder on the reproductive performance of sows also needs to be investigated.
